# Supplementary material for: Mutational Status of SMAD4 and FBXW7 Affects Clinical Outcome in TP53–Mutated Metastatic Colorectal Cancer
Source: Cancers (Basel). 2022 Nov 30;14(23):5921. doi: 10.3390/cancers14235921 (PMC9735648; doi:10.3390/cancers14235921)
Supplement: Supplementary file 1 [file cancers-14-05921-s001.zip › Lahoz et al_Supplementary data.pdf]

Supplementary Data for:

Mutational status of *SMAD4* and *FBXW7* affects clinical outcome in *TP53*-mutated metastatic colorectal cancer

By Lahoz et al.

Supplementary Figure Legends

Figure S1: Flow-chart identifying the number of mCRC patients receiving each treatment approach.

Figure S2: Tree plot displaying the univariate risk of death for all clinical variables included as co-variates in Cox regression models. *P*-values were obtained by the log-rank test, and hazard ratios and 95% CIs using univariate Cox models with proportional hazards for OS. Bold font indicates significance.

Figure S3: Oncoprint plot on the mutational spectrum of the Oncomine Solid Tumor 22-gene panel in mCRC according to primary tumor location: left versus right-sided colorectal tumors. Each row represents a gene and each column an individual tumor sample. Colored bar plots (top) provide data on tumor-related and treatment characteristics annotated in legend (bottom).

Figure S4: Mutational profiling of right colon, left colon and rectum tumors. Bar-plot showing the mutational frequencies for the most altered genes. Left colon includes descendent colon and sigma. *, $P < 0.05$; **, $P < 0.01$

Figure S5: Prognostic value of combined mutated *TP53*/*SMAD4* and *TP53*/*FBXW7* in the mCRC MSK-IMPACT cohort. Kaplan-Meier estimates displaying the cumulative proportion of individuals who were death-free (Y-axis) over the study period (X-axis), and Cox-derived resulting statistics, stratifying by the presence or absence of coexistent mutations in **(A)** *TP53*/*SMAD4* and **(B)** *TP53*/*FBXW7* in the study population with available survival data (N = 797/1,095, 73%) analyzed with the MSK-IMPACT panel-based sequencing. *P*-values were obtained using the log-rank test and hazard ratios using a Cox proportional hazards model. mv., multivariable.

Figure S6: Schematic representation of the machine learning-based approach to assess the predictive value of the annotated clinical variables and mutated genes.
