# Supplementary figures and images for: Mutational Status of SMAD4 and FBXW7 Affects Clinical Outcome in TP53–Mutated Metastatic Colorectal Cancer
Source: Cancers (Basel). 2022 Nov 30;14(23):5921. doi: 10.3390/cancers14235921 (PMC9735648; doi:10.3390/cancers14235921)

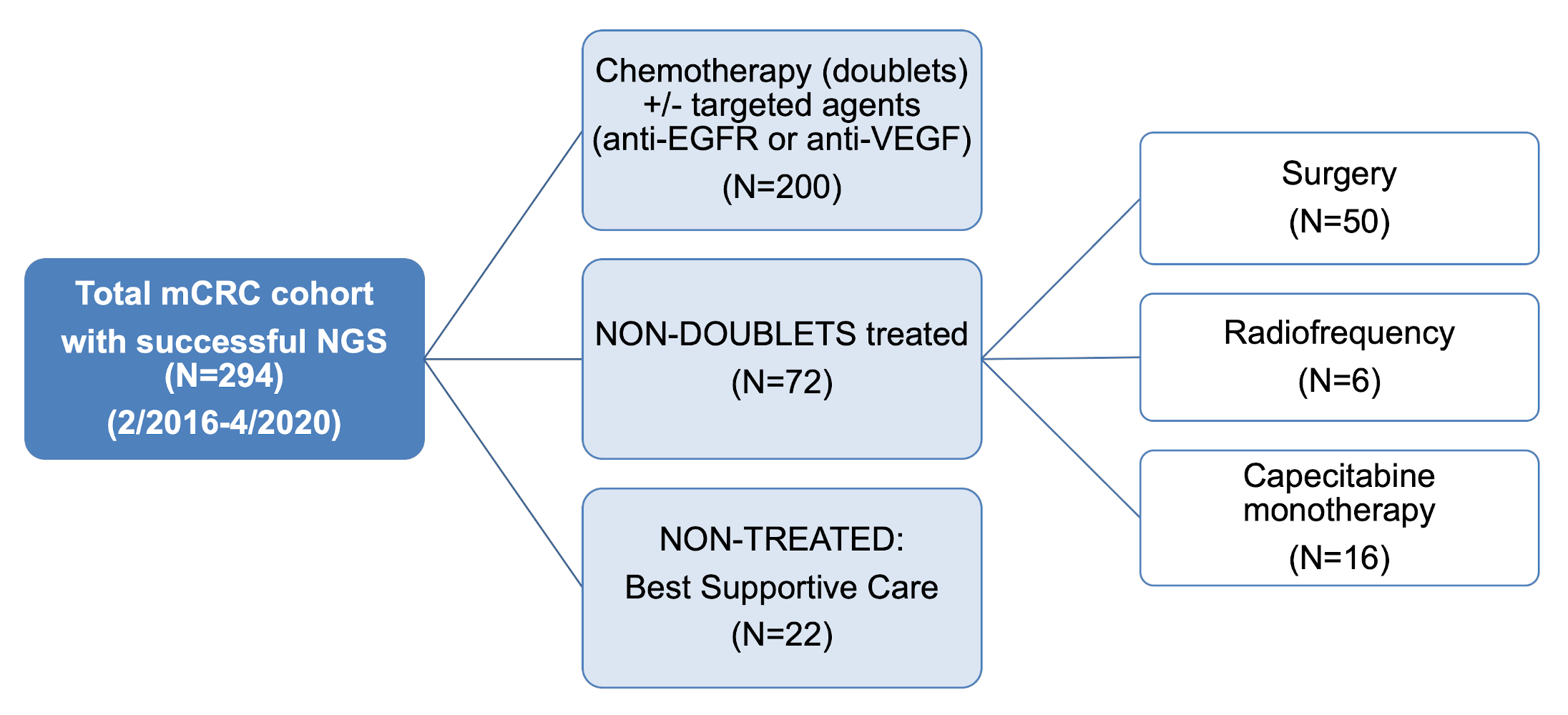

Supplement: Supplementary file 1 [file cancers-14-05921-s001.zip › Lahoz et al_Figure S1.tif]

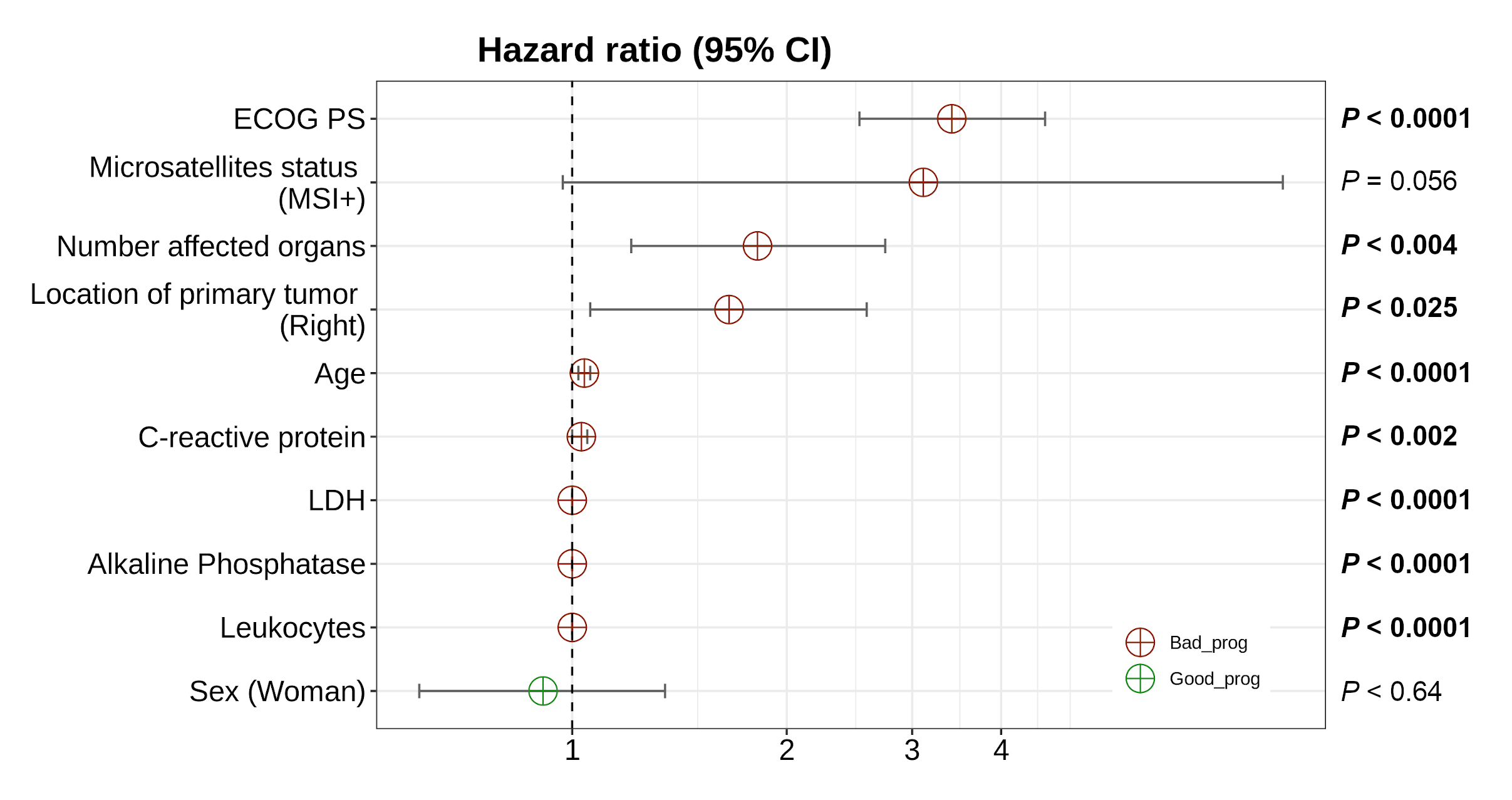

Supplement: Supplementary file 1 [file cancers-14-05921-s001.zip › Lahoz et al_Figure S2.tif]

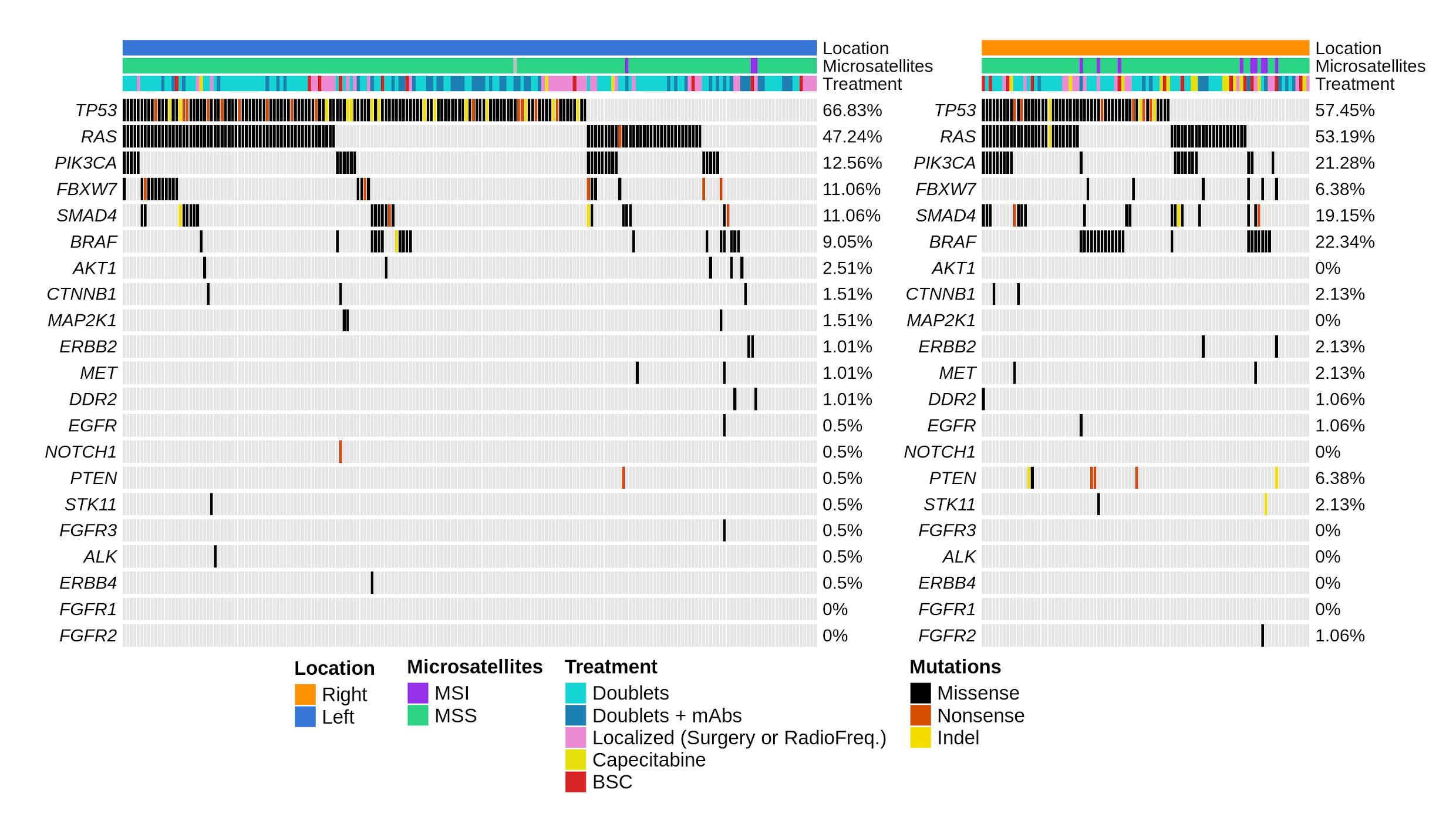

Supplement: Supplementary file 1 [file cancers-14-05921-s001.zip › Lahoz et al_Figure S3.tif]

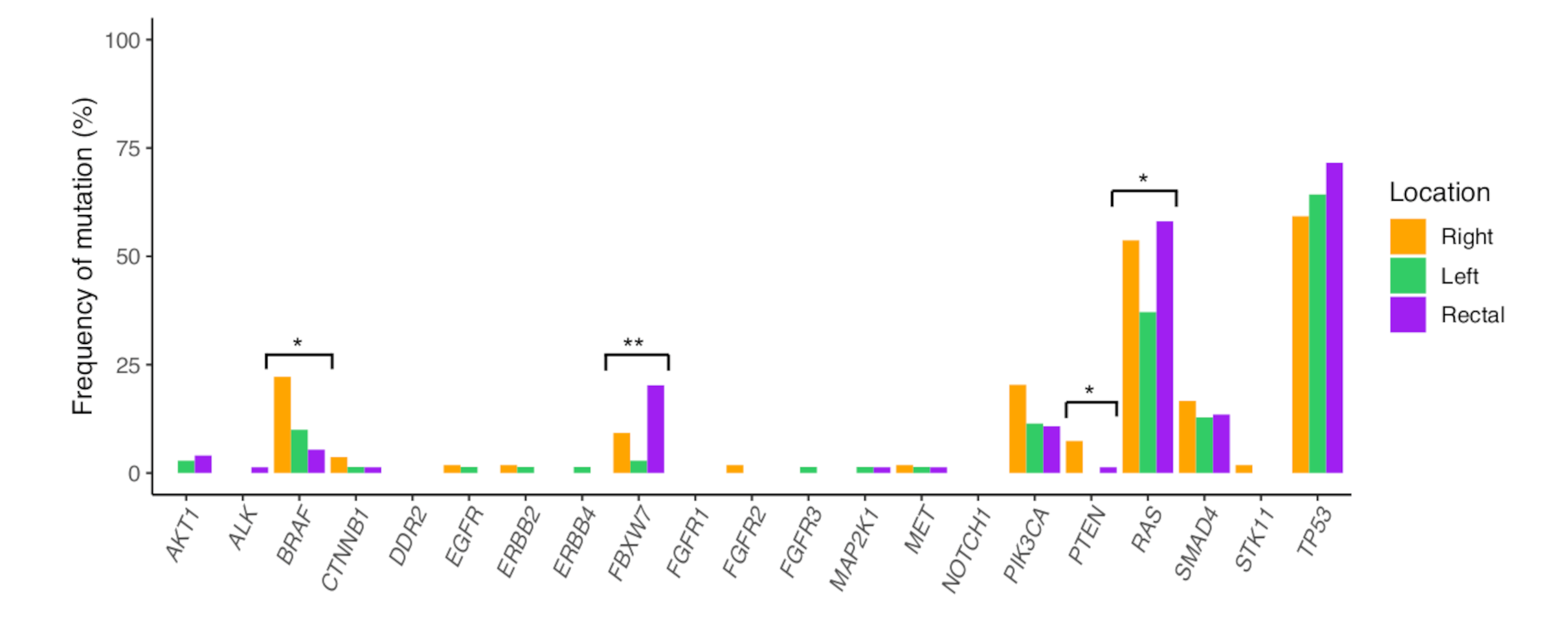

Supplement: Supplementary file 1 [file cancers-14-05921-s001.zip › Lahoz et al_Figure S4.tif]

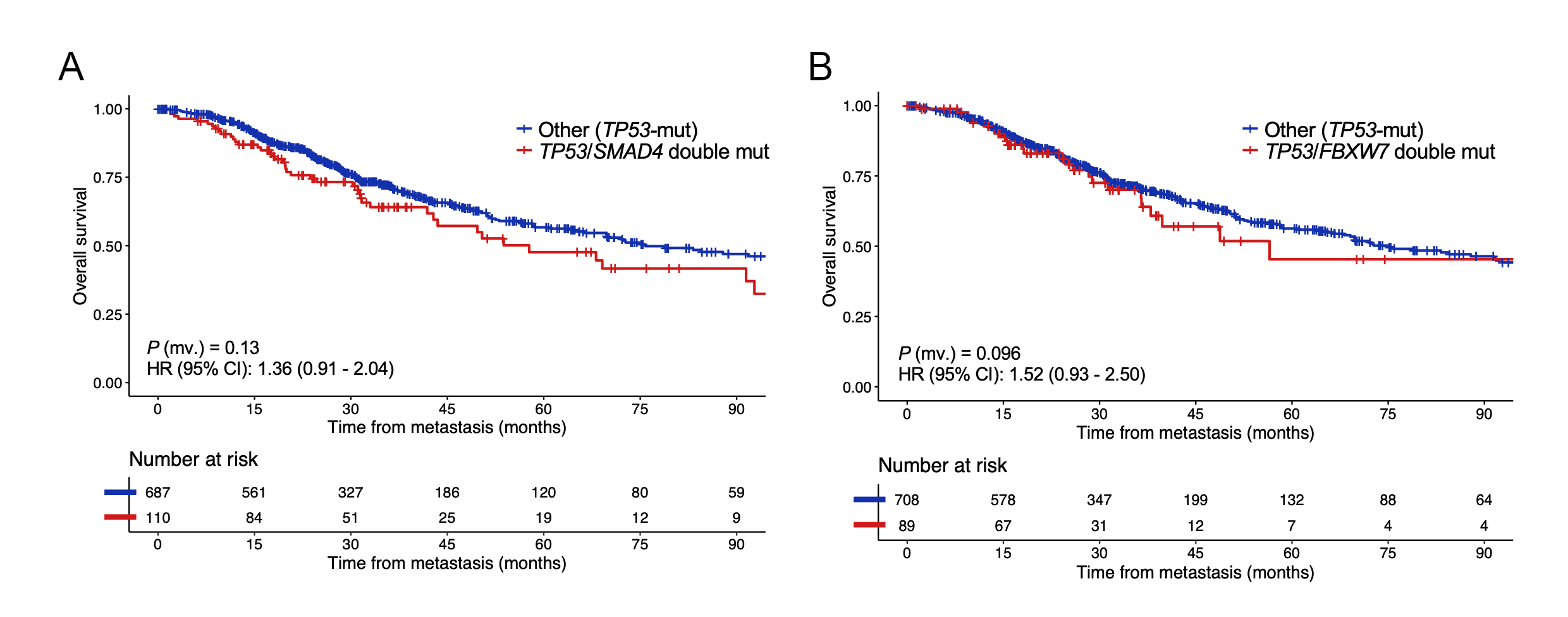

Supplement: Supplementary file 1 [file cancers-14-05921-s001.zip › Lahoz et al_Figure S5.tif]

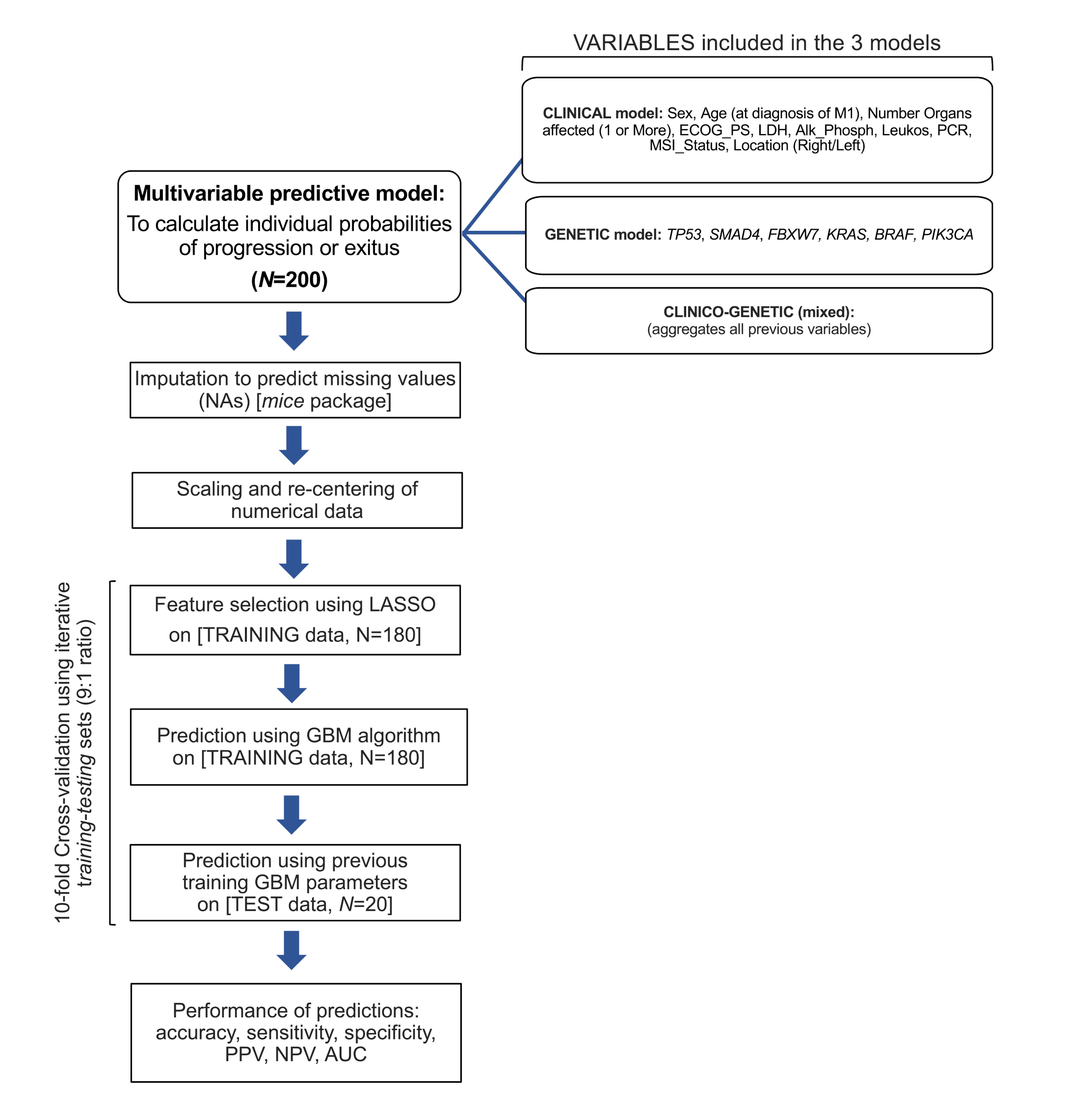

Supplement: Supplementary file 1 [file cancers-14-05921-s001.zip › Lahoz et al_Figure S6.tif]
